# Respiratory Disease: Stormy Outlook for Asthma

**DOI:** 10.1289/ehp.116-a424a

**Published:** 2008-10

**Authors:** Carol Potera

Conventional wisdom holds that rainy days aid asthmatics by washing away pollen pollutants that trigger attacks. But a new study shows that in some cases just the opposite is true—in a report published in the July 2008 issue of *Thorax*, the number of people seeking help at emergency rooms for asthma attacks routinely increased within hours of thunderstorms striking.

Since the 1980s, studies in Canada, Europe, and Australia have documented spikes in asthma cases after thunderstorms. The new study “confirms the association between thunderstorms and outbreaks of asthma in the largest database to date,” says Christine A. Rogers, an assistant professor of environmental health science at the University of Massachusetts, Amherst.

In the current study, a team of climatologists and epidemiologists from two Georgia universities evaluated data from 10 million emergency room visits to 41 Atlanta hospitals over the period from 1993 to 2004. Of 215,832 asthma emergency room visits, they found that 24,350 took place on the day following a thunderstorm, which worked out to about 3% more visits on days after thunderstorms than on other days.

Although a 3% rise may seem small, it could have a significant public health impact for areas with populations in the millions, says study leader Andrew Grundstein, a climatologist at the University of Georgia, Athens. Moreover, “emergency room visits represent an extreme outcome,” notes coauthor Stefanie Sarnat, an epidemiologist at Emory University. Probably many more flare-ups of asthma occurred that were not captured under the study’s criteria, because the people did not seek medical help for asthma symptoms. An average of 70 emergency room visits for asthma are recorded daily in the Atlanta area, according to Sarnat, and the region experiences an average of 50–60 days with thunderstorms.

The counter-intuitive finding of an increased risk of asthma is believed to result from pollen grains swelling and rupturing upon contact with rainwater. The released particles are tinier, more readily inhaled into the airways, and easily spread by gusty winds from thunderstorm downdrafts. “People with asthma and allergies should stay indoors during and after thunderstorms and keep medications close by,” advises Sarnat.

Reports such as a study by Robert J. Trapp and colleagues in the 11 December 2007 *Proceedings of the National Academy of Sciences* predict that rising temperatures and humidity due to global climate change could increase the frequency of severe thunderstorms, which, in turn, could aggravate asthma symptoms. “It’s important for people to know that thunderstorms are another environmental factor that can [exacerbate] asthma,” Grundstein says.

The team has applied for funding to conduct a more detailed analysis using sophisticated tools such as Doppler radar to identify key elements of thunderstorms and meteorological factors that may impact asthma, such as rainfall rates, strength of downdraft winds, and lightning. “Down the road, we may be able to develop a forecast system to warn people who are especially vulnerable to keep them out of emergency rooms,” Grundstein says.

## Figures and Tables

**Figure:**
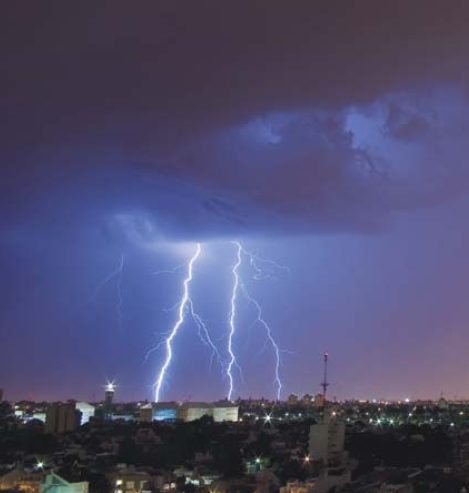
Emergency room visits for asthma symptoms rise in the hours after thunderstorms.

